# Structural Heterogeneity and Quantitative FRET Efficiency Distributions of Polyprolines through a Hybrid Atomistic Simulation and Monte Carlo Approach

**DOI:** 10.1371/journal.pone.0019791

**Published:** 2011-05-24

**Authors:** Martin Hoefling, Nicola Lima, Dominik Haenni, Claus A. M. Seidel, Benjamin Schuler, Helmut Grubmüller

**Affiliations:** 1 Theoretical and Computational Biophysics Department, Max Planck Institute for Biophysical Chemistry, Göttingen, Germany; 2 Department of Biochemistry, University of Zurich, Zurich, Switzerland; 3 Institute of Molecular Physical Chemistry (MPC), Heinrich Heine University, Düsseldorf, Germany; Université d'Evry val d'Essonne, France

## Abstract

Förster Resonance Energy Transfer (FRET) experiments probe molecular distances via distance dependent energy transfer from an excited donor dye to an acceptor dye. Single molecule experiments not only probe average distances, but also distance distributions or even fluctuations, and thus provide a powerful tool to study biomolecular structure and dynamics. However, the measured energy transfer efficiency depends not only on the distance between the dyes, but also on their mutual orientation, which is typically inaccessible to experiments. Thus, assumptions on the orientation distributions and averages are usually made, limiting the accuracy of the distance distributions extracted from FRET experiments. Here, we demonstrate that by combining single molecule FRET experiments with the mutual dye orientation statistics obtained from Molecular Dynamics (MD) simulations, improved estimates of distances and distributions are obtained. From the simulated time-dependent mutual orientations, FRET efficiencies are calculated and the full statistics of individual photon absorption, energy transfer, and photon emission events is obtained from subsequent Monte Carlo (MC) simulations of the FRET kinetics. All recorded emission events are collected to bursts from which efficiency distributions are calculated in close resemblance to the actual FRET experiment, taking shot noise fully into account. Using polyproline chains with attached Alexa 488 and Alexa 594 dyes as a test system, we demonstrate the feasibility of this approach by direct comparison to experimental data. We identified *cis*-isomers and different static local environments as sources of the experimentally observed heterogeneity. Reconstructions of distance distributions from experimental data at different levels of theory demonstrate how the respective underlying assumptions and approximations affect the obtained accuracy. Our results show that dye fluctuations obtained from MD simulations, combined with MC single photon kinetics, provide a versatile tool to improve the accuracy of distance distributions that can be extracted from measured single molecule FRET efficiencies.

## Introduction

Since the development of the Resonance Energy Transfer theory by Förster (FRET) in the late forties [1], and the definition of this technique as a “spectroscopic ruler” in biological systems by Stryer and Haugland [2], single molecule detection [3]–[5] and time-resolved experiments [6] have opened up a new window to probe inter- and intramolecular distances and motions. In a typical experiment, donor molecules are excited by a laser pulse, and part of the excitation energy is transferred to nearby acceptor molecules. The transfer efficiency
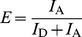
(1)is measured via the donor fluorescence intensity 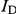 and the acceptor fluorescence intensity 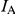. Among other factors, 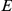 depends on the distance 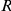 between the donor and the acceptor fluorophores, as well as on the mutual orientation of their respective transition dipole moments. After orientational averaging, the distance dependency is described by Förster's approximation,
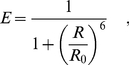
(2)where 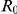 is the so-called Förster radius which denotes the distance at which 50% of the donor excitation is transferred to the acceptor molecule.

This relation is widely used to monitor structural changes in biomolecules via FRET efficiency measurements [2], [7]. To that aim, donor and acceptor fluorophores are covalently attached to specific sites of the macromolecule of interest. Taking into account the flexibility of the fluorophores and their linkers, the measured intensities provide information on the mutual distance of these specific sites [8]–[11]. The use of multiple dye pairs allows for triangulation of biomolecules, which provides three-dimensional structural information [10], [12]–[16].

In single molecule setups, distributions and distance fluctuations of individual molecules are accessible [4], [17]–[19]. If the scatter of the observed efficiency distributions in these experiments is broader than the expected shot noise, distance distributions can be estimated [20]. For distance changes in the biomolecule, which are slow compared to the burst duration, time resolved information is then accessible [21], [22]. By recording millisecond fluorescence bursts while the molecules diffuses through a confocal laser volume, conformational motions in the same time scale have been resolved [21], [23], [24].

FRET spectroscopy has proven particularly successful in situations where the mutual orientation distribution of the transition dipole moments can be considered isotropic and uncorrelated. Examples are freely diffusing dyes, or dyes attached to flexible and solvent-exposed parts of a protein [18] or nucleic acids [10], [11]. In this case, orientational averaging gives rise to the well-known orientation factor 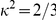, which is by convention included within the Förster radius 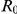 [7]. In contrast to this average 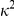, the instantaneous orientation factor 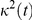 can assume values in the range of 0 to 4.

Particularly when triangulating biomolecules, however, the dye motion is often far from isotropic due to steric restrictions set by the biomolecule, as well as due to electrostatic or hydrophobic interactions between the dye and the protein surface [25]–[30]. Since the mutual dye orientation is typically inaccessible to experiments, the 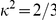 approximation provides only qualitative insights, unless the free and rapid reorientation of the dyes is commonly verified by fluorescence anisotropy measurements [31]. For this reason, efficiency distributions rather than distances are often reported.

The orientational dynamics uncertainty of fluorophores has been addressed via several routes. Empirical, semi-empirical, and theoretical models [32]–[36] for the orientational factor have been developed, assuming that the dynamics of the dyes can indeed be described by a time average. Recent computer simulations [37], [38] have suggested that the mutual dye orientation can be highly anisotropic, with 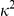-values deviating markedly from 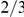 (0.24–1.02 [38]; 0.71–2.81 [37]). 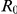 has been refined through fluorescence quenching measurements of multiple fluorophores [39].

Despite these efforts, three main problems remain. First, the assumption of an isotropic dye orientation distribution is invalid or difficult to establish in most cases [40], [41]. Second, possible correlations between the distance and dye orientation distribution are neglected in the above treatments [38]. Third, the orientational sampling during individual bursts may be incomplete, in which case the dye distribution relevant for the observed efficiency depends on the duration of the bursts. In all three cases, applying an average 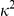 – as opposed to the 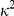 of instantaneous and time-dependent Förster transfer rate coefficients – leads to an additional broadening of the efficiency distribution [25], and biased distance distributions are obtained.

To overcome these limitations, we have developed an approach that combines molecular dynamics (MD) simulations of a dye-labeled biomolecule in solution with Monte Carlo (MC) simulations of dye excitation, FRET transfer, and fluorescence decay events. This approach involves four steps.

First, extended and fully atomistic MD simulations of the solvated biomolecule, labeled with a FRET dye pair, serve to cover the biomolecular dynamics at the fluorescence decay time scales of the system. To capture structural motions that are slower than the nanoseconds time scale accessible to MD simulation, several MD trajectories are recorded starting from different isomers and combined into a comprehensive ensemble using appropriate Boltzmann weights.

In the second step, time-dependent mutual dye orientations extracted from these trajectories are recorded. These orientations are then used to derive time-dependent instantaneous resonance energy transfer rate coefficients 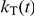. Within a short time interval 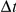, these rate coefficients specify the probability 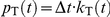 that a FRET transfer event takes place, for each instant of time.

In the third step, using 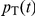, a large number of MC runs is carried out to simulate and collect many individual photon absorption and excitation, FRET transfer, and emission events. For each photon absorption event, an instant of the trajectories is chosen randomly, and the probabilities are propagated appropriately until a photon emission or radiationless decay event occurs. After averaging over sufficiently many events, fluorescence intensities 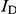 and 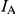 are calculated. The numbers of photos recorded from the donor and the acceptor dyes, respectively, finally determine an average FRET efficiency value 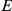. Similar approaches using dye conformations from simulations have been proposed recently [42]–[45].

To mimic single molecule FRET (smFRET) experiments, in a fourth step the emitted photons are collected into bursts according to the experimental photon burst size distribution (BSD). The efficiency in each burst is then calculated, and efficiency histograms are obtained, similar to single molecule experiments. By construction, this procedure takes shot noise accurately into account.

This hybrid simulation approach will enable one to calculate efficiency distributions that can be directly compared to measured efficiency distributions. Vice versa, we will develop a systematic approach to reconstruct distance distributions by combining the dye orientation and photon statistics at hand with measured efficiency distributions.

**Figure 1:**
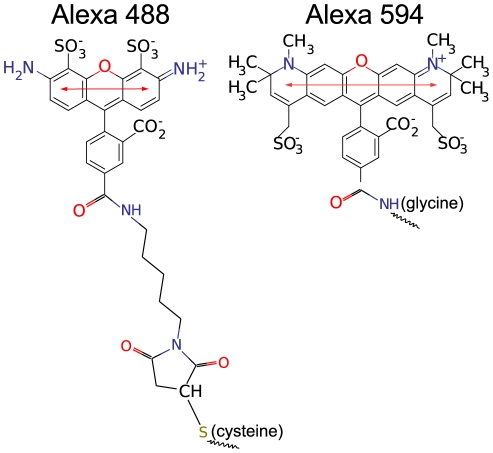
Dye and Linker Structures. Structure and transition dipole moments of Alexa 488 and Alexa 594. The red arrows show the orientation of the transition dipole moments. MarvinSketch was used to draw the chemical structures, Marvin 5.3.0.2 , 2010, ChemAxon (http://www.chemaxon.com).

**Figure 2:**
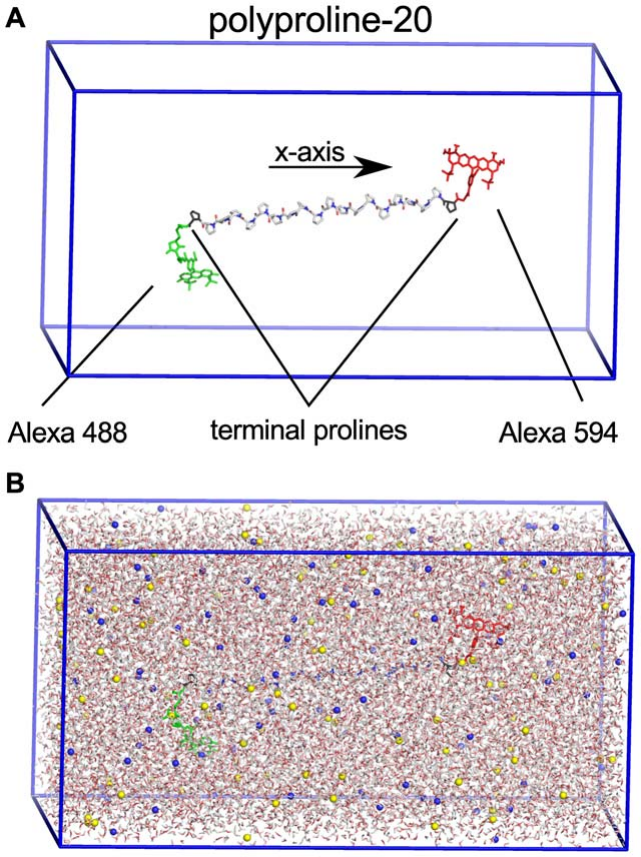
System Setup. (A) All-*trans* polyproline-20 molecular structure including Alexa 488 (green) and Alexa 594 (red) dyes attached by their corresponding linkers. The simulation box is shown in blue, terminal prolines used to restrain the position are depicted in black. (B) Fully solvated system is shown including 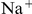 (blue) and 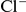 (yellow) ions.

Here we apply this approach to a polyproline 15, 20, and 30-mer [46] with two FRET dyes (Alexa 488 and 594, Fig. 1) attached to both termini [2], [31], [45](Fig. 2A). As dye-labeled polyproline chains have been widely used as “rigid rods” to test the validity of the approximations underlying Förster's theory, and to gauge the Förster radius of several of FRET pairs in different environments [2], [31], [45], much of the current understanding relies on the particular properties of these systems. Initially assumed to be quite rigid, all-trans polyproline helices were used in the definition of FRET as a “spectroscopic ruler” [2]. This assumption was challenged quite early [47], [48], suggesting that polyproline chains exhibit a substantial degree of flexibility [49]. The issue is still not fully resolved.

For these reasons, polyproline flexibility has been revisited recently by performing single molecule FRET recordings [31], [50] and simulations [45] on these molecules. Indeed, unexpectedly broad efficiency distributions were seen, suggesting substantial structural heterogeneity. A detailed analysis of single molecule data showed the heterogeneity that persists on time scales greater than 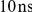 [50]. Recent NMR experiments [45] pointed to a considerable population of *cis*-isomers within all-*trans* polyproline helices, which might contribute further to the structural flexibility and heterogeneity of polyprolines. These findings put the suitability of these molecules as “rigid rods” in question, and the unexpected complexity of their dynamics requires a detailed study of the structural ensemble in solution at room temperature.

Here we attempt a comprehensive characterization of the polyproline structural heterogeneity by combining atomistic simulations with single molecule FRET data. Resting on a direct comparison of single burst efficiencies collected over many bursts, our approach is based on much fewer assumptions than the standard interpretation of FRET experiments. In particular, this approach includes 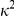 averages, on the basis of the detailed molecular dynamics of the system, and cases where the motion of the dyes is slower than the donor fluorescence decay time are readily handled. Moreover, all possible correlations between the dye movement and the distances are included, such that accurate mutual orientation distributions are obtained. Finally, the approach fully accounts for the photon count shot noise. Vice versa, comparison with experiments will enable us to test our approach. As we will demonstrate, our approach serves to combine dye orientational dynamics from MD with experimental FRET efficiency distributions at increasingly refined approximation levels.

The good agreement of distance distributions of polyproline obtained by this approach with the reference distribution suggests that this combination allows extraction of improved quantitative geometrical information from single molecule FRET experiments. By comparison with synthetic FRET data, the validity of the reconstruction will be established.

## Methods

### System Setup

The studied system comprises a polyproline peptide of 15, 20 or 30 proline residues [46], an amino-terminal glycine and a carboxyl-terminal cysteine residue, to which a succinimide ester and maleimide derivatives of Alexa 594 and Alexa 488 dyes [52](Fig. 1), respectively, are attached. Figure 2A shows the simulation system for the polyproline-20 [53] within a rectangular simulation box. Figure 2B depicts the box filled with explicit water molecules and 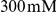 NaCl, corresponding to the ionic strength of 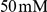 sodium phosphate buffer used in the experiment [31]. The number of 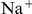 and 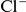 ions was chosen such as to obtain a neutral system.

In aqueous solution the most stable configuration for polyproline chains is the polyproline II (PPII) helix [53], [54], characterized by dihedral angle values 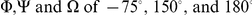, respectively [47], with the *trans*-isomer as the most favorable configuration. Nevertheless, in water a marked fraction of *cis* peptide bonds the PPII helices is observed. By NMR experiments a fraction of approximately 10% for proline at the C-terminus of the chain and 2% within the chain was measured [45], with *trans* to *cis* transition times of 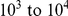 seconds [50], [55]. As this is far beyond MD time scales, separate simulations were performed for all relevant isomers, for subsequent weighted averaging. To this end, all possible isomers containing one single *cis* peptide bond were considered, i.e., 20 *cis*-trajectories for the polyproline-20 with dyes attached. Additionally, for polyproline-30, a subset of 61 isomers with *cis*-bonds at two positions was simulated.

### Force Field

For water molecules, the TIP4P model was employed [56]. Force field parameters for the peptide were taken from a modified OPLS-AA force field [57] including custom parameters for the two dyes and their corresponding linkers. Alexa 488 and Alexa 594 are highly conjugated systems whose parameters are not included within the standard OPLS-AA force field. Figure 1 depicts the atomic structure of the two dyes together with the orientation of the transition dipole moments. All dye parameters (bonded and Lennard-Jones) – except for the partial charges – were assigned via an analogy approach from similar OPLS-AA groups [58].

Because FRET occurs when the donor dye is in the excited state and the acceptor in the ground state, partial charges of these corresponding states were used in all our simulations for the dyes. The fact that the partial charges calculated for the ground and excited states differed only by a small amount suggests that the effect of this simplified treatment on the dynamics of the dyes is small. All partial charges were calculated by fitting to the electrostatic potential surfaces (EPS approach [59]) obtained from *ab-initio* B3LYP Density Functional Theory (DFT) calculations with the 6-31G* basis set. All *ab-initio* calculations were performed with the GAUSSIAN 03 program package [60]. First, for reference, the point charges for the 20 natural amino acids were calculated with B3LYP/6-31G* CHelpG population analysis to assure compatibility of the derived charges with OPLS-AA. A mean scaling factor of 0.9 was calculated by averaging the multiplicative factors of each amino-acid, which minimizes the mean square deviation between OPLS-AA and DFT charges (amino-acid scaling factors shown in Suppl. Table S1).

For the ground state of the two dyes, the same protocol was used. For the excited state, we determined charge differences with respect to the ground state for each atom in two steps. First, point charges were determined from Configuration Interaction Singlets (CIS) calculations for the first excited state using the STO-3G basis set. From these values, in a second step, point charges were subtracted, that were obtained from Hartree Fock (HF) calculations with the same STO-3G basis.

For both, ground and excited state, the charges were averaged to reflect the internal symmetry of the molecule, and scaled with the previously calculated scaling factor of 0.9. Finally, a small offset was added to all partial charges to re-establish the correct total charge of the system.

### Molecular Dynamics Simulations

All MD simulations were carried out with the GROMACS 4.0.7 simulation software package [61]–[63]. Each proline system was energy-minimized by steepest descent to convergence. Periodic boundary conditions were applied in all three dimensions. V-Sites on hydrogens [64] were used allowing 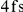 integration time steps. After minimization, 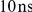 equilibration simulations were performed. From the last 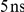 of these simulations, starting conformations for all subsequent production runs were selected at random instances (Table 1). Solvent and ions as well as the solute were separately coupled to an external temperature bath with a time constant of 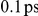 applying the v-rescale algorithm [65], [66]. The system was coupled to an isotropic pressure bath of 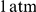 using the Parinello-Rahman algorithm [67] and a time constant of 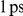. Bond lengths were constrained to their equilibrium lengths with LINCS [68]. The cut-off for Lennard-Jones interactions was set to 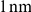. Electrostatic interactions between charged groups at distances below 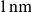 were calculated in direct space, while for the long-range interactions the particle-mesh-Ewald method [69] with a grid spacing of 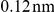 and fourth order spline interpolation was used. All simulations were performed with random Maxwell-distributed starting velocities at 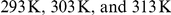 (Table 1).

**Table 1:** Performed molecular dynamics simulations.

Proline Length	Isomer	Temperature	Number of simulations
		K	
pro15	all-*trans*	293	10
pro15	single-*cis*	293	30
pro20	all-*trans*	293	20
pro20	all-*trans*	303	10
pro20	all-*trans*	313	10
pro20	single-*cis*	293	40
pro30	all-*trans*	293	10
pro30	single-*cis*	293	30
pro30	double-*cis*	293	61

Simulations are listed according to isomer and applied temperature. Single-*cis* simulation were carried out for all possible *cis*-isomer positions. For polyproline-30, in addition, a representative set of 61 isomers, randomly picked from the 870 possible isomers with two *cis* bonds, was simulated. All simulation lengths are 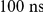 summing up to a total sampling of 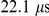.

Soft restraints were imposed to suppress rotation of the entire molecule in the box and thus to allow the use of a small simulation box, adapted to the shape of the molecule. To this end, the component of the difference vector perpendicular to the x-axis (Fig. 2A) between the centers of mass of the two terminal prolines was restrained to zero with a weak harmonic potential (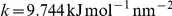, corresponding to a Boltzmann distribution of width 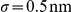). We assume that these soft restraints leave the internal dynamics of the molecule unperturbed.

### Resonance Energy Transfer Rates

All FRET efficiencies were calculated from the MD simulations using following kinetics,
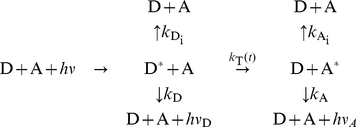
(3)


Starting after a photon adsorption event by the donor dye, this kinetics is described by
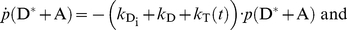
(4)

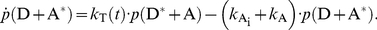
(5)


In Eq. 3, 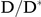 is the donor (Alexa 488) and 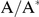 is the acceptor (Alexa 594) dye in their ground and the excited state, respectively. 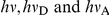 denote the exciting photon and photons emitted by the donor and the acceptor dye. The rate coefficients refer to FRET (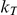), fluorescence and internal conversion of the donor (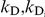), and fluorescence as well as internal conversion of the acceptor dye (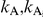).

The rate coefficients were calculated from the lifetimes 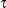 of the dyes and their respective quantum yields 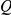,
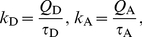
(6)

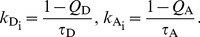
(7)


For the Alexa 488 and 594 dyes attached to polyproline peptides, we used the measured lifetimes 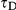 of 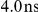 and 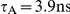. To obtain photon statistics directly comparable to the experiment, the quantum yields were combined with the detector efficiencies into (relative) effective quantum yields using the correction matrix defined in Ref. [70]. In this framework, 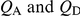 correspond to the diagonal correction matrix elements. For the simulations, we averaged the two detector channels used in the experiment, yielding 0.77 and 1.0 for donor and acceptor effective quantum yields, respectively. Crosstalk, direct acceptor excitation, and background were found to change the photon statistics only by a small amount and thus are neglected in our MC approach.

For the time-dependent FRET rate coefficient 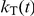, which depends on the electronic coupling between the two dyes and thus also on their mutual orientation at each instant, we used Förster's dipole approximation for the electronic coupling,
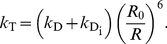
(8)


In Eq. 8, 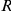 is the distance between the geometric center of the ring system of the acceptor and the donor dyes, and 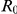 is the Förster radius (the distance of 50% excitation transfer), which is proportional to the time-dependent orientation factor 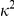,
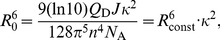
(9)where 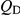 is the quantum yield of the donor in the absence of the acceptor, *J* the spectral overlap integral (Franck Condon factor), 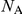 Avogadro's number, 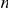 the index of refraction of the solvent, and 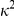 is the time-averaged orientation factor [3], [70], [71]. For the pair Alexa 488 – Alexa 594, a Förster radius 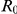 of 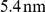 has been determined [7], [72], based on the assumption of isotropic dye orientations i.e., 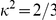. To describe time-dependent Förster transfer, 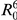 in Eq. 8 is therefore replaced by 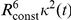, with 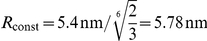.

**Figure 3:**
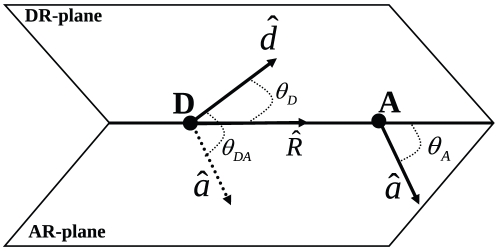
Geometry of dye orientations. Three angles define the orientation factor 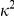, the angle 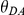 between 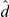 and 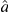, and the angles 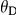 and 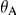 between 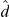 and 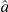, respectively, and 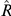. The DR and DA plane are defined by 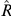 and 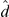 as well as 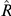 and 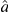.

The orientation factor
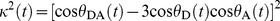
(10)depends on the three relevant angles defined in Fig. 3. The transition dipole moment orientations within the molecular frame of the dyes were chosen parallel to the ring system plane, and connecting the terminal rings of each dye (Fig. 1) [73].

Using the above framework, for all MD trajectories orientation factors 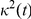 and distances 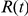 were calculated and stored for each time step, thus obtaining time-dependent FRET rate coefficients 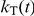, which will be used below. Supplementary Video S1 shows distance, orientation factor and transfer efficiency for an exemplary trajectory.

We note that for small inter-dye distances (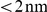), when terms of higher order than the dipolar are not negligible, Eq. 8 can be replaced by multipole expansion of the coupling potential or the transition density cube method [42], [74] in a straightforward manner, such that accurate FRET rate coefficients are also obtained in these cases. In the present work, the dipolar coupling potential was used.

### Single Photon Generation

**Figure 4:**
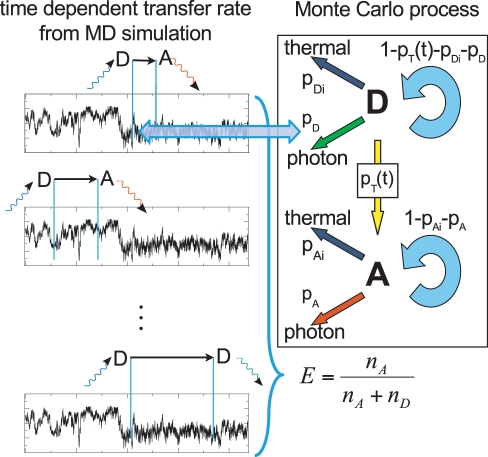
Photon generation by Monte Carlo. FRET transfer rate coefficient vs. time, calculated from a molecular dynamics simulation (box, left part). A random starting point on the trajectory is chosen at which the donor dye is assumed to be excited by a photon (blue). Then, for each time step the MC process on the right side is evaluated according to the corresponding probabilities until de-excitation occurs. Four de-excitation pathways are considered, thermal de-excitation of donor or acceptor (dark-blue) and respective photon emissions (donor: green; acceptor: red). The ratio of the collected donor and acceptor photons is used to calculate a FRET transfer efficiency.

For direct comparison with smFRET burst counts, we developed a Monte Carlo (MC) procedure to calculate single burst FRET efficiencies from 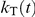. In the experiments, the arrival times of individual photons from single molecules were recorded. Accordingly, and following the kinetics scheme Eq. 3, multiple individual photons were generated in a Monte Carlo process (Fig. 4). For each photon, we proceeded as follows.

First, a random donor excitation instance was chosen from a randomly chosen trajectory (Fig. 4 left). Next, the Markov scheme in Fig. 4 (right) was iterated in time steps of 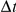 until either photon emission or radiationless decay occurred (see Suppl. Video S2). In the latter case, the MC run was discarded, in the former, the photon (donor or acceptor) was recorded. During each MC cycle and using an integration time step 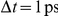, transitions were randomly selected according to probabilities 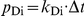 for thermal de-excitation, 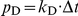 for donor photon emission, 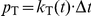 for FRET transfer and 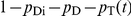 for no state change. Acceptor de-excitation probabilities were calculated in the same way, but with consistent transition probabilities 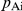 and 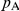, which allowed to skip the remaining Monte Carlo step and to record the emitted photon right away. All random numbers were generated with an SIMD-optimized Mersenne Twister algorithm [75], [76].

In the experiment, no FRET is seen for dyes in or close to van-der-Waals contact, presumably due to quenching by electron transfer [77]. The effect of quenching at low inter-dye distances is not described with Förster theory, and therefore also not in our MC process. To correct for this, photons are rejected if the inter-dye distance is below 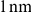 during the photon generation when comparing to experiments.

### FRET Efficiency Calculation

Averaged over many MC runs, the collected de-excitation events 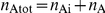 and 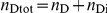 from donor and acceptor, respectively, were used to determine the average efficiency
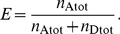
(11)


In experiments, only radiative de-excitation events (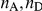) can be recorded. We therefore followed the same way in reconstructing the total number of de-excitation events using the respective fluorescence quantum yields,
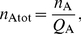
(12)and analogously for 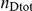.

To directly relate efficiency distributions from MC sampling to single molecule FRET measurements, the effect of shot noise and burst size distribution has to be taken into account properly [31], [78]. Here, a sufficiently large number (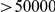) of bursts has been measured, which provided sufficient statistics such that the experimental burst size distribution was used for combining the MC generated photons into bursts. After correction for quantum yield and detector efficiency, for each burst a single FRET efficiency value was calculated using Eq. 11. Collecting FRET efficiencies from many bursts yielded efficiency distributions that can be directly compared to the measured ones. As in the experiment, only bursts larger than 100 photons, after correction for the effective quantum yield, were used.

### Inclusion of *cis*/*trans* isomer heterogeneity

So far, we have considered only one isomeric state of the proline polymer, e.g., the all-*trans* state. As has been found by NMR, however, each peptide bond undergoes isomerizations, with a small but non-negligible population in the *cis*-isomer, and with a larger *cis*-population for the terminal peptide bond at the C-terminus [45]. Because the isomerization times of minutes to hours are much longer than all other relevant time scales, we considered a weighted ensemble of all possible relevant isomerization states and performed the above MD and MC simulations with efficiency calculations separately for each isomer. Subsequently, employing 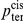 and 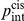 from NMR experiments [45] as probabilities for the occurrence of *cis*-isomers for C-terminal and internal peptide bonds, receptively, a weighted average was obtained (Table 2).

**Table 2:** Isomer weights.

Isomer	Probability
all-*trans*	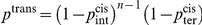
#1 cis, other trans	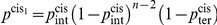
#2 cis, other trans	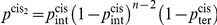
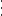	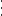
#n-1 cis, other trans	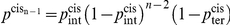
#n cis, other trans	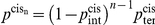

Here, 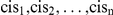 indicate the position 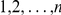 of the *cis* peptide bond in the chain, starting from the amino terminus.

### Single-Molecule Experiments

Peptide samples were prepared as described previously [31]. Single-molecule fluorescence experiments were performed with a MicroTime 200 confocal microscope (PicoQuant, Berlin, Germany) equipped with a pulsed 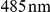 diode laser (LDH-P-C-485B, PicoQuant) and an Olympus UplanApo 60 x/1.20 W objective. After passing through a 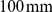 pinhole, sample fluorescence was separated by a polarizing beam splitter cube into components parallel and perpendicularly polarized with respect to the excitation light. Subsequently, both components were further divided into donor and acceptor photons by means of dichroic mirrors (585DCXR, Chroma), filtered (donor emission filters: Chroma ET525/50 M, acceptor emission filters: Chroma HQ650/100), focused on avalanche photodiodes (PerkinElmer Optoelectronics SPCM-AQR-15), and the arrival times of all detected photons were recorded using suitable counting electronics (Hydra Harp, PicoQuant, Berlin, Germany).

## Results and Discussion

Time-dependent conformations of the two dyes and their mutual orientations for the three polyproline systems considered here (Fig. 2) were obtained from multiple 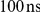 MD trajectories of the all-*trans* and *cis*-isomers. MD simulations totaling 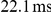 were carried out for the different isomers, chain lengths, and temperatures (Table 1). We first focus on polyproline-20 in the all-*trans* isomer as the most stable configuration in water and analyzed two main factors relevant for the FRET efficiencies, the distance 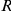 between the two fluorophores and the orientation factor 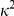.

### Distance Distributions

**Figure 5:**
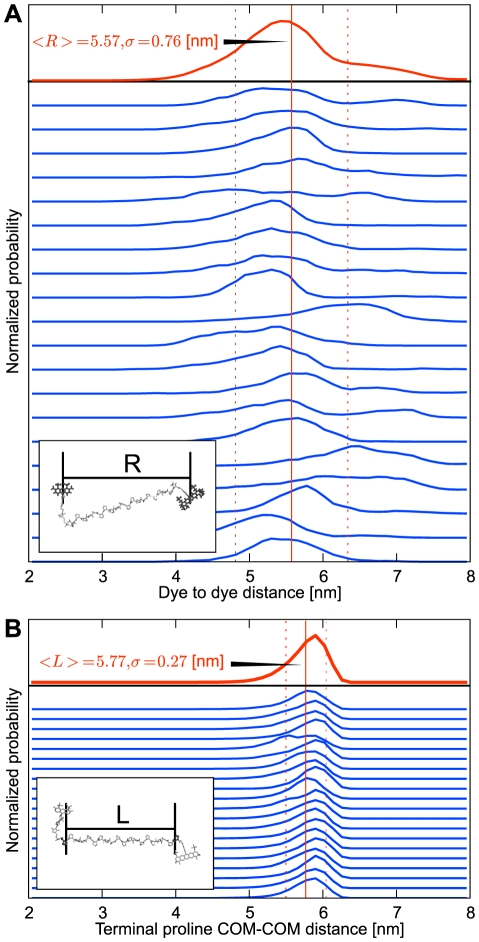
Distance distributions. (A) Histograms of the distances between the geometric centers of the ring systems of the two dyes for 20 all-*trans* MD simulations. (B) The distance histogram between the COM of terminal prolines from the polyproline-20 chain, for the same simulations. The insets visualize the measured distance in each plot. Respective averages are shown in red; vertical lines denote the mean and standard deviation.

Fig. 5A shows the distributions of dye-to-dye distances (defined by the geometric center of the ring system) from individual 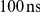 simulations. The fact that the distributions differ from each other shows that the individual simulations are not fully converged to represent the full all-*trans* ensemble. To improve convergence, multiple simulations were combined. The apparent differences between the individual distance distributions are mainly due to slow transitions between subpopulations of dye-conformations (for more details see section “Preferred Dye Conformations”).

To better characterize the subpopulations and how they differ between the individual trajectories, the distance between two terminal proline residues center of mass (COM) was analyzed. As shown in Fig. 5B, these length fluctuations are much smaller compared to the dye-to-dye distances. In addition, the mean length of individual simulations shows only small variations.

These small length fluctuations point to considerable rigidity of the polyproline peptide, which indeed originally motivated its use as a molecular ruler. From the angular fluctuation 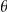 of selected segment pairs, separated by length 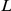, a persistence length 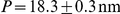 was obtained via
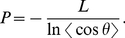
(13)


Here, 3 proline residues (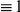 PPII helix turn) defined a segment and its tangent with a segment length of 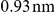. The all-*trans* chains are indeed quite rigid and do not strongly deviate from the type II helix structure model.

Because of the stiffness of the polyproline, the observed broader distribution between the dyes mainly originate from the flexible dye linkers rather than from the flexibility of the polyproline chain.

### Orientational Dye Dynamics and Orientation Factor 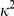


**Figure 6:**
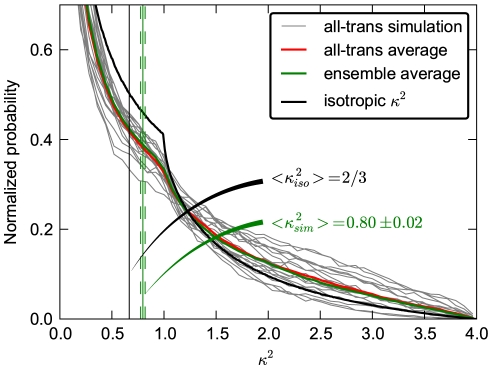
Distributions of the orientation factor 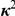. Each gray line shows to an orientation factor histogram from one of the 20 all-*trans* simulations at 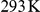, with the average shown in red. The green curve (full ensemble) additionally includes the *cis*-isomers with appropriate weights, the green vertical line shows the corresponding average and its statistical error (dashed). The black curve shows the 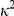 histogram for an isotropic dye orientation distribution, with the well known mean value of 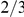 (vertical black line).

Figure 6 shows the 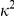 distributions derived from 20 all-*trans* simulations (gray) as well as their average (red). For comparison, an isotropic 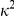 distribution is shown (black). As shown, the individual simulations scatter considerably, with respective mean 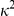 values between 0.58 – 1.06. Averaging over all 20 simulations, the mean 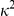 of all-*trans* simulations was 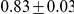, and 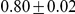 for the complete ensemble including all *cis*-isomers (Fig. 6). Both values agree within statistical error and significantly deviate from the isotropic 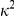 value of 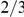.

As seen from the 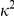 histograms of individual MD simulations, the sampled dye geometries differ for each simulation, which underscores the importance of averaging multiple simulations. The obtained more realistic 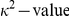 shifts the effective Förster radius from 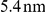 to 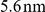.

Next, we determined the correlation between 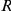 and 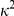 for the 20 all-*trans* simulations and found a mean Pearson correlation coefficient of 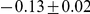. Because 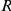 and 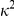 are assumed to be uncorrelated in Försters RET theory, this finding suggests that using a distance-dependent 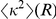 might further improve the distance reconstruction, as will be discussed below.

Table 3 shows mean auto-correlation times of different variables from the simulations (exemplary autocorrelation plot shown in Suppl. Fig. S1 ). The orientation factor 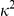 shows the fastest decay (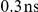), whereas the terminal orientation and the dye to dye distance are in the ns regime (Table 3) and thus comparable to the donor fluorescence decay times. Calculated fluorescence anisotropy decay timescales [34], [35] of 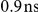 in our simulations agree with experimentally measured decay times of 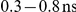 [31] within the accuracy of the simulation [34] and thus indicate a correct modeling of the dye dynamics by our force field.

**Table 3:** Time scales of motions.

	mean	SEM	min	max
	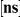	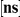	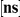	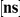
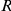 (dye-to-dye)	2.96	0.52	0.71	8.68
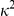 (orientation factor)	0.34	0.04	0.15	0.86
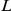 (terminal prolines)	0.80	0.20	0.30	4.12
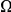 (terminal orientation)	4.96	0.86	1.06	14.55
Anisotropy decay (Alexa 488)	0.90	0.08	0.42	1.66

Autocorrelation times of all-*trans* polyproline-20 with their respective standard error of the mean (SEM), minimum and maximum. Terminal orientation 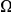 denotes the autocorrelation times of the cosine of the angle between the terminal proline tangent vectors.

These autocorrelation times determine the correlation of the dye conformations and distances as probed by successive photons and, therefore also, how many structures probed by each burst are effectively statistically independent. Further, this autocorrelation time may determines the size of the sub-ensemble of conformations that is actually probed by FRET, because the fluorescence intensities of the two dyes also depend on past transfer efficiencies. We will therefore examine the influence of these effects on the quality that can be achieved for the distance reconstruction described further below.

### Preferred Dye Conformations

**Figure 7:**
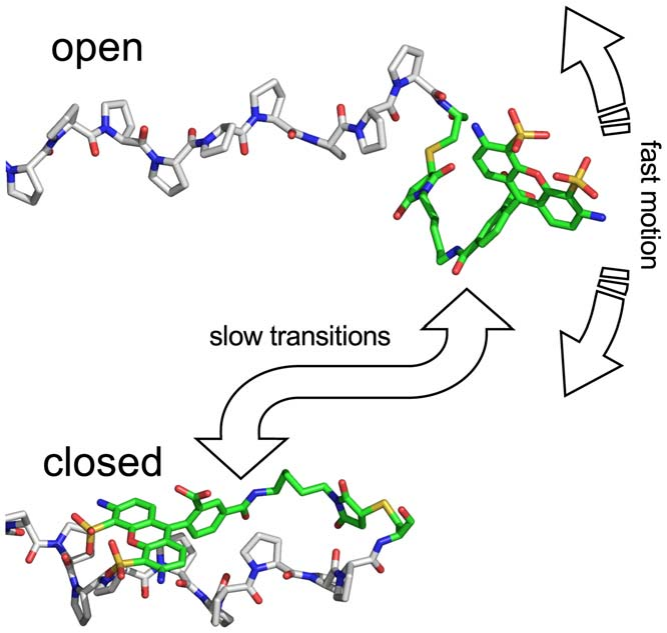
Conformational heterogeneity of Alexa 488. Several conformations of the Alexa 488 dye and its linker attached to the proline chain during MD simulations are seen in the simulations. For the *open* conformation, fast large amplitude motions are seen for the dye whereas hydrophobic interactions restrict the dye mobility in the *closed* conformations (one representative example is shown). Additionally slow transition between the open and closed conformations are seen.

What is the structural origin of the orientation factor 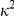 deviation from its isotropic value of 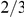
? A closer inspection of the MD simulations revealed that hydrophobic interactions of the dye linker with the proline chain enhanced the population of certain conformational sub-states, similar to previous reports[45]. This effect is more pronounced for Alexa 488 due to the longer linker. For Alexa 488, two distinct conformation sub-states (*open* and *closed*) were seen (Fig. 7).

To test the stability of these confomer ensembles, we analyzed distances and the orientation factors of the all-*trans* polyproline-20 system at elevated temperatures (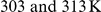, Table 1). No significant impact on the values for 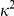 was found (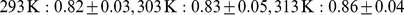). Also the dye-to-dye distance 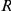 showed no systematic trend towards open or closed conformations (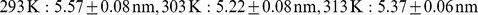). For the polyproline-20 chain length 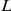 (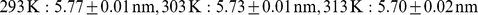), a small decrease with increasing temperature was seen. In summary, the applied temperature changes neither seem to significantly influence the population ratios of the two conformations, nor the relative dye-to-dye orientations. However, due to the limited sampling, we cannot fully exclude small effects, which may arise at larger temperature changes. It will be an interesting challenge for future experimental work to directly identify the presence of the dye conformations observed here, e.g. from a broadening of fluorescence anisotropy distributions in single molecule experiments [79], or from the effect of measurements under conditions that increase the solubility of the fluorophores on the transfer efficiency histograms.

### Efficiency Distributions from Individual Simulations

**Figure 8:**
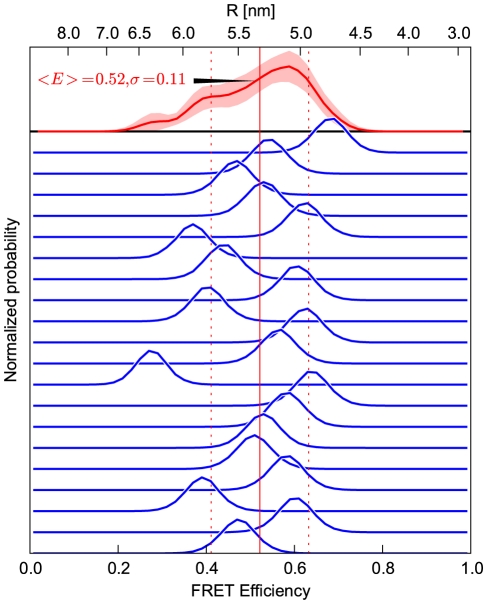
Spread of the efficiency amongst individual simulations. Transfer efficiency histograms (blue) obtained via MC sampling from 20 all-*trans* MD simulations of polyproline-20 at 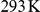. The red curve at the top depicts the efficiency combined from all 20 trajectories, where each burst is still combined from photons of one trajectory; the bootstrapping standard error, calculated from 100 random samples, is indicated by the shaded area. The vertical lines indicate the mean efficiency and its standard deviation.

Figure 8 shows FRET efficiencies calculated separately from all 20 all-*trans* MD simulations. As already expected from the dye-to-dye distance distributions, also the mean FRET efficiencies cover a broad range from 0.27 to 0.66 with 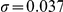 to 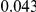. These standard deviations 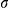 were compared to the expected shot noise 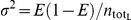 [80], [81] for each simulation mean efficiency 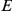 using the lower experimental BSD limit (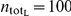) resulting in a width 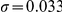 to 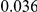. Thus, the efficiency peak observed in the individual traces of our simulations is mainly broadened due to the photon shot noise.

Comparison of the distance distributions (Fig. 5A) with the efficiencies (Fig. 8) illustrates the effect of signal averaging over an entire fluorescence burst, subsequently referred to as '‘burst averaging’. To see this, consider naive transformation from distances to efficiencies using Eq. 2, which would result in much broader efficiency distributions than those observed in Fig. 8. This narrowing is due to the combination of multiple photons, and thus also of distances, into one burst, such that each efficiency value represents a corresponding average [11]. It is this averaging, which markedly narrows obtained efficiency distributions and also obscures much of the structure seen in the distance distribution.

### Isomeric Heterogeneity

To account for the isomeric heterogeneity due to the presence of *cis*-isomers, which reduce the average distance between the two dyes [45], additional MD simulations for all possible isomers were performed (Table 1). Using the population estimate of Table 2, the full ensemble includes 5%, 8%, and 15% of isomers with more than one *cis* bond for polyprolines of length 15, 20, and 30, respectively. Thus, for proline 15 and 20, we included only the single-*cis* conformers within the ensemble. For polyproline-30, estimating the impact of multi-*cis* isomers, additionally a subset of double-*cis* isomers was considered (Table 1). In the isomer simulations, all the other bonds were kept in the *trans* configuration, and the same MD parameters and protocol as for the all-*trans* isomer were used. FRET efficiencies were then calculated as explained before.

**Figure 9:**
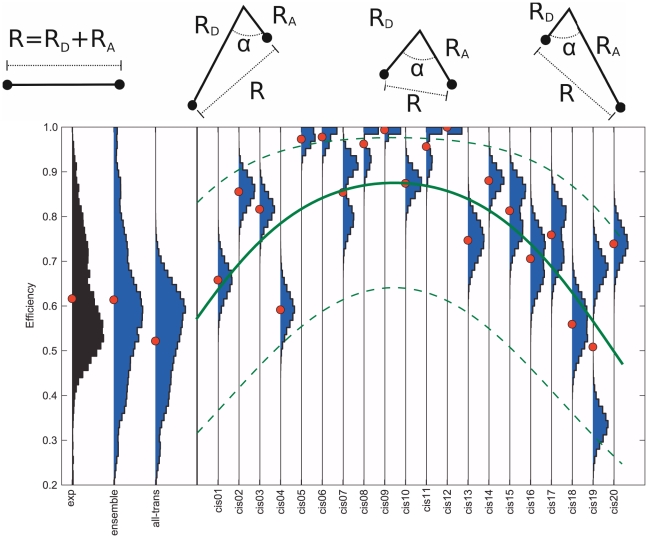
FRET efficiency of *trans* and *cis* isomers. Comparison between measured FRET efficiency histograms (black) and histograms computed from the simulations (blue: ensemble, all-trans and cis01 ... cis20). Red dots denote the respective mean values. The simple model sketched on top and defined in the text describes the general trend (green line) that isomers with a *cis*-bond close to the termini show lower efficiencies, whereas those with *cis*-bonds close to the polymer center tend to yield higher efficiencies. The dashed green lines estimate the spread of the average efficiencies of the *cis* simulations mirroring the spread found for the all-*trans* simulations (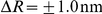). For illustration purposes, the photons of the individual *cis* were not discarded when generated below 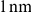 as described in the Methods Section. The high efficiencies observed for *cis*-6 to *cis*-12 result from dyes in contact and are quenched in the experiment.

Figure 9 shows FRET efficiency distributions and averages for the all-*trans* and *cis* polyproline-20 chains in comparison with experiment. As expected, the average efficiencies of the *cis*-chains are larger than that of the all-*trans* isomer, due to the reduced distance of the terminal prolines. The largest reduction is seen for *cis*-bonds in central positions, thus attributing measured high efficiencies to those isomers. This behavior can be captured in a simple model (Fig. 9, top), in which the *cis*-isomer is described by a kink angle 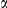 between the two stiff parts of the molecule, with distances 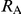 and 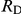 between the *cis*-bond and the respective termini, and 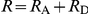 being the all-*trans* distance between the two termini. 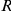, was determined from the all-*trans* mean efficiency using Eq. 2 and split up on 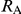 and 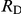 for each *cis* isomer according to the *cis*-bond position. To account for the distance change due to the linker and the observed dye conformations (Fig. 7), an offset 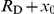 and 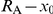 was allowed for as an additional fit parameter. After fitting to the model to the average *cis*-efficiencies using Eq. 2, an angle of 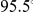, and and offset 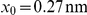 was obtained. The resulting model is shown as green line in Fig. 9 and has to be compared to the mean efficiency values (red dots). The dashed line shows an offset of 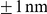 in efficiency space as error estimate. The offset towards Alexa 594 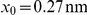 agrees with the deviation of the average dye-to-dye distance from the proline length (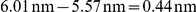) within the accuracy of this simple model.

Next, ensemble efficiency distributions were calculated by combining *cis* and *trans* isomers according to their population in solution. Using the population of individual isomers as determined by NMR [45], 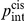 and 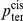, weights were determined as listed in Table 2. For polyproline-20, these weights are 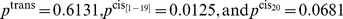.

For poly-15 and polyproline-30, the same 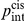 and 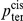 measured on polyproline-20, were applied assuming that they are not strongly influenced by the proline chain length. Because the *cis*-content is larger in polyproline-30, an error in 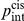 and 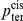 has a larger impact on the accuracy of the ensemble composition. For example, if polyproline-30 has a 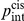 value of 4% instead of 2%, the multi-*cis* isomer ensemble content increases from 15% to 37%, whereas the all-*trans* isomer contribution drops from 50% to 28%. As a result, the obtained ensemble efficiency histograms sensitively depend on the value of 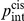 and 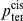 , particularly for the longer polyproline-30 chain.

As seen before, the *cis* ensemble content and thus the content of isomers with double-*cis* bonds increases with the chain length. For polyproline-30, this contribution is about 15%. To estimate the impact of double-*cis* species on the efficiency histogram, we simulated a subset of double-*cis* isomers (Table 1). The obtained weights for each chain length and isomer were used in the next step, to calculate efficiency distributions of the entire ensemble.

### Combining Photons into Bursts

So far, we calculated efficiency distributions of single simulations (Fig. 8) and their accumulated histograms (Fig. 9). To calculate burst efficiencies in closer resemblance to single molecule experiments, we need to define how the recorded photons are combined, e.g. from multiple trajectories. The specific approach depends on the relative time scales of the relevant processes in the experiment and the simulation. In single molecule experiments on freely diffusing molecules, ten to hundreds of photons are recorded in each burst of several ms duration. On the simulation side, in contrast, multiple 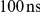 trajectories are available. We consider three different ways of combining photons into bursts and compare the resulting efficiencies to experiment.

The relevant time scales are the two autocorrelation times for the dye dynamics, namely those of the orientation factor 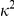 and of the distance 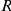 fluctuation, from hundreds of picoseconds (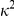) to ns (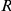) (Table 3); further the polyproline chain dynamics of a specific isomer with the slowest motions in the 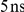 range (Table 3, 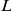 and 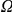), the *cis* to *trans* isomerization time ranging from minutes to hours for polyproline [82], the experimental burst recording duration of several ms and the respective inter photon times [83], as well as the simulation trajectory length of 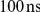.

**Figure 10:**
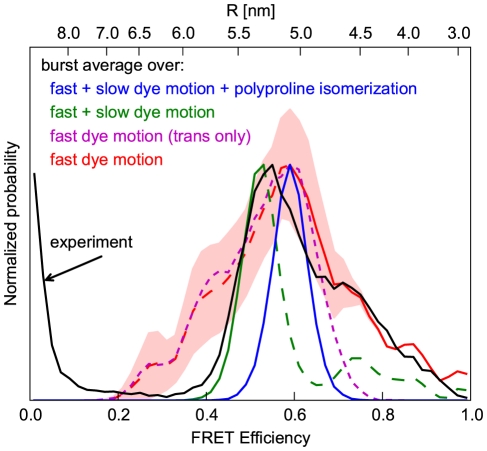
Combining photons into bursts. Comparison of different photon accumulation methods for a full polyproline-20 ensemble at 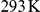 with the experiment (black). Three different accumulation methods (colors) were considered. First, efficiencies were calculated from the full ensemble (blue), for which each photon burst has been combined from photons of all *cis* and *trans* simulations, and which therefore average over all motions and heterogeneities covered by the simulations. Second, each efficiency value was calculated from photons of all simulations of a randomly chosen isomer (green), thereby averaging over all dye motions but not over different isomers. Third, each efficiency value is derived from photons of one single trajectory, and weighted by the appropriate ensemble probability (red). The impact of the *cis*-isomers is demonstrated by comparison to the all-*trans* only efficiency histogram (magenta). The bootstrapping standard error (Fig. 8) of the all-*trans* isomers is drawn as light red area. Efficiency histograms were normalized to their maxima.

In the first case (burst average over fast and slow dye motions as well as the polyproline isomerization), the burst duration is assumed to be longer than all other time scales mentioned above. Accordingly, in this case, each measured burst consists of photons from the entire isomeric ensemble. To achieve a most comprehensive sampling, therefore, photons from all available trajectories with their appropriate ensemble weight are combined. The blue line in Fig. 10 shows the resulting efficiency distribution as a single peak whose width is solely determined by the shot-noise. Experiments measuring ensemble efficiencies (e.g., CW in bulk) correspond to this case, except that in ensemble measurements an effectively infinite number of photons is gathered, and therefore the shot noise vanishes. For the polyproline system at hand, however, the isomerization times are long compared to the burst duration, and thus this case is not expected to apply here. Indeed, the measured efficiency distribution (Fig. 10, black) is much broader.

Accordingly, for the second case (burst average over fast and slow dye motion), we assume that the isomerization time is longer than the average burst duration, with the remaining dye and chain dynamics still being fast compared to the burst duration. In this case, all photons from a measured burst originate from one particular isomer. Because the trajectory length is much shorter than the burst duration, each burst is generated from all trajectories of a particular isomer. Figure 10, green line, shows the resulting efficiency distribution. Because in contrast to the previous case, averaging is not done over multiple isomers within each burst, as assumed above, the individual *cis* isomers contribute high efficiencies (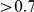) to the efficiency distribution (Figure 10). As shown in Fig. 10 (dashed green line), these high efficiencies are also observable in the experiment (black line). In addition, Fig. 10 reveals that the low efficiency side agrees with the experimental distribution (solid green line). However, when comparing the region around 0.7, a gap between the all-*trans* peak and the high efficiency *cis* region is present, not found in the experiment. In analogy to the comparison of this case and the above case, which averages over the polyproline isomerization, this hints at additional dynamics slower than the burst duration, averaged out in the current case.

If this is true, one would expect a better agreement for the third case considered here. In this case (burst averaging over fast dye motion only), we now assume that the dye dynamics contains additional components that are slow compared to the burst duration. An example of such a component is the transition between different conformations of the dye, e.g. the ones shown in Fig. 7. Therefore, all photons in a burst originate from a distinct dye conformation with an interconversion time larger than 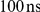. In resemblance to this, each burst is generated from one distinct simulation trajectory. The previous assumption of slow isomerization times compared to the burst recording duration is automatically contained in this case, since each trajectory contains a single distinct isomer. Figure 10 (red line) shows the resulting efficiency distribution. In contrast to the burst average over fast *and* slow dye motion, where all-*trans* and *cis*-isomers were resolvable (Fig. 10, green line), the conformational heterogeneity on time scales beyond 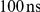 and thus of different simulations, is now visible as already observed in Fig. 8. As shown in Fig. 10 (red line), this heterogeneity is particularly pronounced for the all-*trans* simulations due to the largest number of simulations (Table 1) and the all-*trans* isomer being the largest fraction of the ensemble. The small numbers of simulations result in a considerable statistical error, shown as red area in Fig. 10 and calculated from the all-*trans* isomer. When comparing this result to the experiment, the high efficiency side (solid red line) with *cis*-efficiencies agrees with the experiment (black). The discrepancy (gap around 0.7) previously observed (burst average over fast *and* slow dye motion) vanishes. However, an additional low efficiency shoulder is visible not present in the experiment (dashed red line).

This deviation is not within statistical uncertainty (Fig. 10, red area) and may be due to several reasons. First, because all simulations have been started from the *open* conformation (Fig. 7), this conformation may have been oversampled. Second, although the dye dynamics described by the fluorescence anisotropy decay times agrees with the experiment, we cannot fully exclude over- or underestimation of the dye-hydrophobicity with our choice of partial charges. Third, this discrepancy can be explained by the presence of two different dye dynamics in the experiment as described below.

Overall, the low-efficiency side (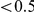 in Fig. 10) in case of burst averaging over fast *and* slow dye motions agrees well with the experiment, whereas on the high efficiency side (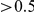), better agreement is seen for burst averaging over fast dye motions only (Fig. 10, solid green and blue vs. black). From the above discussion of time scales, this finding would imply that the low efficiency side (i.e., large distances) is governed by fast dynamics, whereas parts of the slow dynamics govern the high efficiency (i.e., shorter distances) side only. Close inspection of our simulations suggests a possible structural explanation for this finding. In particular, the hydrophobic interactions between the polyproline and the Alexa 488, which give rise to the structural heterogeneity shown in Fig. 7, with very slow transitions between the *open* and *closed* conformation. In the *open* conformation, the dye-reorientation is fast compared to the burst duration and thus sampled within a single burst, in agreement with the low efficiency side (Fig. 10). In the *closed* conformations, the dye dynamics is largely restricted, with the high FRET efficiency therefore being governed by the slow transitions between these sub-states, in agreement with the observed burst averaging over fast and slow dye motions.

**Figure 11:**
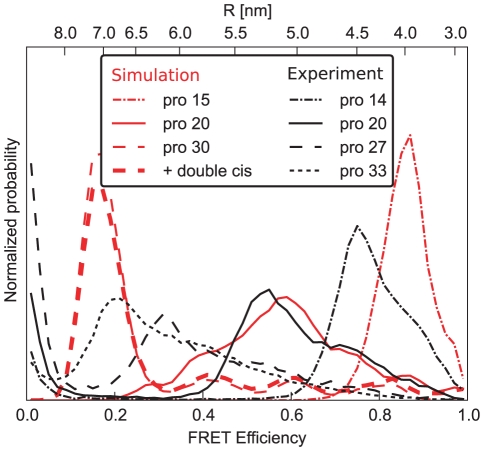
Comparison between proline 15, 20 and 30 and experiment. Efficiency histograms averaging over fast dye motions only (Fig. 10) are shown for three different polyproline-lengths (red, dashed-dotted, solid, dashed), and corresponding measured efficiency distributions (black). For polyproline-30, inclusion of double-*cis* isomers (see Text S1) only slightly changes the efficiency histogram (bold dashed line).

Next, we compare efficiency distributions for different dye-labeled proline lengths. Figure 11 shows the calculated efficiency distributions (burst averaging over fast dye motions only) from simulations with proline lengths 15, 20, and 30 (solid lines) as well as measured efficiencies for lengths of 14, 20, 27, and 33 (dashed). The general length effect, increase in efficiency for shorter prolines and vice versa, is observed.

For polyproline-15, the calculated distribution has the same narrow shape as found in the experiment, however with the simulated efficiency distribution shifted towards higher efficiencies. Purely from the length difference between polyproline-14 (experiment) and polyproline-15 (simulation), an opposite shift is expected. A similar slight discrepancy is seen for polyproline-30, where the peak should be located between the experimental peaks of polyproline-27 and -33, but is seen in Fig. 11 somewhat below polyproline-33.

While the overall agreement between simulation and experiment is good, this observed systematic deviation is striking. Apparently, compared to our simulation results, the experimental efficiencies tend to be shifted slightly towards 0.5 within both the high as well as the low efficiency regime. Overall, such behavior cannot be explained by an uncertainty in the measured 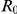, which would lead to a uniform shift in one direction. With the same argument, also force field inaccuracies, which might, e.g., overestimate the hydrophobicity of the dyes and thus also the population of the *closed* conformation, are incompatible with the observed deviation. As a possible explanation one might consider a modified Förster law with, e.g., an effective power smaller than 6 in Eq. 2 (e.g. a power of 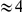 yields the best agreement of the simulated and experimental peak positions). Such effects have been observed previously [31] and may originate from inter-dye quenching or the breakdown of the point dipole approximation [41], [84]. As a third possible cause, decreased fluorescence lifetimes at high efficiencies leading to a stronger deviation from 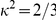 has been discussed [31], but is already included within our simulation approach and thus unlikely to explain the deviation.

Comparing the shapes of the polyproline-30 curves, both the calculated as well as the measured efficiency distributions share shoulders reaching into the high efficiency regime. However, this shoulder is much more pronounced in the experiment than in the simulation. Closer inspection shows that the shoulder originates exclusively from *cis*-isomers. To interpret this discrepancy it is thus helpful to ask what fraction of the *cis*-population, according to the NMR results, is expected to fall into this high efficiency range. Interestingly, with the 2% *cis*-population (per bond) from NMR, and considering the fact that only about 2/3 of the cis-population contributes to the high efficiency shoulder (whereas about 1/3 contributes to the main, all-*trans* peak, see Suppl. Fig. S2), the NMR results are incompatible with the high (ca. 50%) population seen by FRET. Accordingly, a small correction of the NMR values towards higher populations of the *cis*-isomers would resolve both the discrepancy between NMR and FRET as well as that between FRET and our calculated efficiency histogram. In contrast, our neglect of multiple *cis*-conformers is unlikely to explain the discrepancy, as seen from the small effect when including the double-*cis*-species (Fig. 11, bold dashed red line) as the dominant multiple *cis*-population.

In experiments, a peak around zero efficiency is seen for all proline species. This peak originates from polyproline molecules lacking an active acceptor dye, either because of imperfect labeling or because of photobleaching of the acceptor dye during the measurement [31]. In our simulations, all molecules carry a donor and an acceptor dye, and photobleaching is not considered; the zero efficiency peak is thus absent. The clear separation of the zero efficiency peak from the rest of the signal allows us to compare only the signal from the “intact” molecules with the simulated data.

### Reconstructing Distance Distributions from FRET Efficiencies

We have shown above that accurate efficiency histograms can be calculated from a combination of atomistic MD simulations and Monte Carlo photon sampling. Now we will ask the inverse question: Can the dye orientation distributions obtained from the simulations be combined with *measured* FRET efficiency histograms in such a way as to enable reconstruction of more accurate distances and, possibly, also distance distributions, than by the established 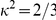 approximation? And if so, which accuracy can be expected at the different conceivable levels of approximations that were mentioned in the Introduction?

To address these questions, the efficiency histogram calculated from the hybrid MD/MC approach (where the distance distribution is known) as well as the single molecule FRET efficiency histogram from the experiment (where the distance distribution is unknown) were used as input for the backward calculations. The thus reconstructed distance distribution, both from the synthetic and the experimental FRET data, were then compared to the known distribution from the simulation. For each level of approximations, thereby, the impact on accuracy of the respective assumptions is quantified.

As a common framework for a proper definition of the applied approximations, we consider the most general (linear) transformation from a distance distribution 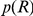 to an efficiency distribution 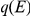 in terms of transfer functions 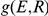,
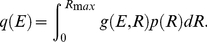
(14)


Each level of approximation, will be defined through an approximately specified transfer function 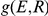 – or, after discretization, transfer matrix. In all cases, the all-*trans* polyproline-20 structural ensembles were used for the calculation of the transfer function as well as to generate the synthetic efficiency distribution 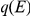; to reconstruct 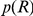 from the experimental efficiency distribution, which involves an isomer mixture, the full structural ensemble with appropriate weights was used to calculate the transfer function (except for transfer functions 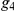 and 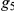). At each approximation level, 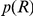 was then reconstructed from 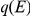 and 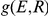 by inverting a discretized version of Eq. 14.

As, generally, such inversion is numerically highly unstable, regularization assumptions are required. Here, motivated from the observation of two structural conformers (*open* and *closed* conformation, cf. Fig. 7), we assumed that 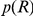 can be described sufficiently accurate by the sum of 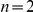 Gaussian functions centered at 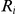 of width 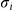,
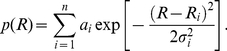
(15)


With this description, the 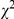 between the calculated and the reference efficiency distribution was minimized by variation of 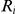 and 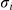 using the two array differential evolution algorithm [85]. Extension of this method to more Gaussian functions or to a more sophisticated model [86] is straightforward.

At the lowest level of refinement, the usually assumed isotropic dye orientation distribution is considered, implying 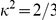, independent of the mutual distance between the two dyes. The efficiency distribution 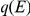 was obtained from the donor-acceptor distance distribution 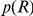 via the usual Förster formula, Eq. 2,
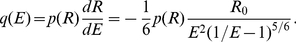
(16)


**Figure 12:**
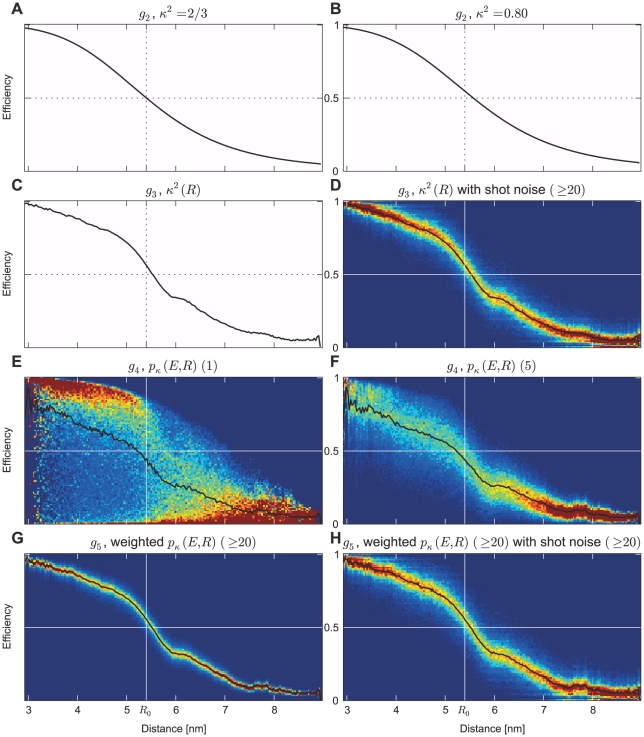
Transfer functions 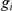 at increasingly refined approximation levels. Transfer functions A, B, and C are shown as black curves; the remaining transfer functions are shown color-coded, with averages highlighted as black curves. Transfer function 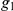 was calculated using the assumption of 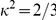 (A). For 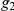, 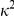 was adjusted to represent the ensemble average in the simulations (B). 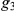 includes the distance dependency of 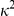 without (C) and with (D) shot noise derived from the experimental BSD (burst size or lower burst size cutoff given in brackets). In contrast to a distance dependent averaged 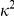, 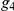 includes the 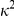 distributions at each distance without (E) and including averaging within a burst (F). In 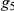, the time dependent photon emission (Fig. 14) is included, shown without (G) and with experimental shot noise (H).

In the more general transfer function formalism used further below (Eq. 14), the above result (Eq. 16) is readily recovered from the transfer function
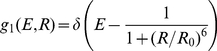
(17) shown in Fig. 12A.

**Figure 13:**
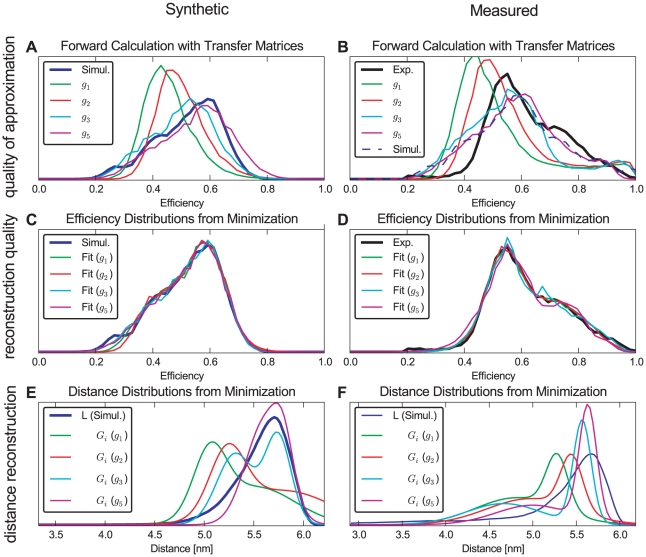
Distance reconstruction from efficiencies. Reconstruction of distance distributions from synthetic efficiencies with known distance distributions from simulations, and measured efficiencies with unknown distance distribution. Two ensembles were considered: The left column consists of all-*trans* polyproline-20 at 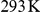. The right column also includes the *cis*-isomers with appropriate weights and uses experimental efficiencies as reference. In the first row (A and B), the efficiency distributions obtained from multiplying the transfer matrices (discretized transfer function) with the distance distributions obtained from simulations are shown together with efficiencies derived from our simulations and experiment as reference. The second row (C and D) depicts the efficiency obtained by optimizing the parameters of two Gaussian distance distributions as a measure for the reconstruction quality. The efficiencies were calculated by multiplying the transfer matrix with the Gaussian distance distributions with optimized parameters (see Text S1). The distance distributions obtained from reconstruction and simulation are shown in the third row (E and F). In all graphs, the reference is plotted with a bold line. Notably the experimental reference distance is inaccessible in F. The employed transfer matrices include experimental shot noise.

Figures 13A and B show how well the respective transfer functions capture the relation between 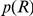 and 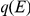 as obtained from the simulations. At this first level of refinement, using the above 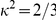 transfer function for both the all-trans ensemble (A) as well as for the full ensemble, containing all isomers (B), quite narrow efficiency distributions (green curves) are obtained, which are also shifted towards lower efficiencies with respect to the reference efficiency distributions (blue, black). As expected, the reconstructed distance distributions, Fig. 13E and F (same color scheme), are also shifted towards smaller distances, with the maximum being off by more than 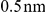. Further, the reconstructed distance distribution has a shoulder that is not seen in the reference distribution. Overall, the reconstruction is not satisfactory at this level of refinement. Figure 13C and D show, that for the all-*trans* MC and the full ensemble experimental efficiencies, respectively, adjusting of parameters in Eq. 15 led to convergence.

To quantify to which extent the assumption of an isotropic dye orientation distribution causes this discrepancy, at a second level of refinement the correct 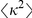 value was used, as obtained from the respective MD simulation ensemble (cf. Fig. 6). Still, this value was assumed independent of the distance between the two dyes. This approximation is described by the transfer function
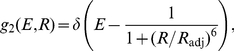
(18)with 
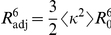
, and 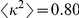. As seen in Fig. 12B, this refinement results in a slight shift of the Förster curve with respect to the isotropic dye orientation approximation (Fig. 12A).

At this improved level of refinement, a slight shift of the calculated efficiency distributions towards the reference distributions is observed (red curves in Figs. 13 A, B). As a result, correspondingly improved reconstructed distance distributions are obtained (Fig. 13 E, F). However, the shapes of the efficiency curves are still too narrow, and the shoulder in the reconstructed distance distribution is still present. Apparently, these artifacts are mainly caused by further approximations not investigated so far.

Therefore, at the third level of refinement, we drop the previous assumption that the dye orientation distribution is independent of the donor-acceptor distance. Accordingly, the MD structure ensemble was split into groups according to mutual dye distance, and an average orientation factor 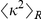 was calculated separately for every group, i.e., as a function of 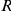. Note that this distance dependent orientation factor
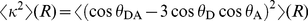
(19)differs from the previous ones in that it captures correlations between the dye orientation distribution and the donor-acceptor distances. This can be used to construct the transfer function
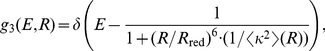
(20)defining 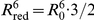.

As seen in Fig. 12C, the resulting transfer function is not strictly monotonic any more, such that the inverse transformation to 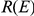 is not straightforward and, the above regularization techniques need to be applied.

This refinement step yields a marked improvement of both peak position and shape of the obtained efficiency distributions (Figs. 13 A and B, cyan). Only a slight peak shift towards lower efficiencies remains for the all-*trans* ensemble (Fig. 13 A), as is also seen for the experimental efficiencies in Fig. 13B. Also for the distance reconstruction, the dominant peak is now at the correct position in both cases (Fig. 13 E, F), although the second peak in the synthetic distance reconstruction using the all-*trans* ensemble still remains and leads to an overestimate of the distribution for smaller distances.

So far, our transfer functions uniquely defined the efficiency 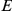 for each distance 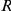. Before continuing with further refinement steps, we demonstrate how the experimental shot noise impacts the reconstruction of distances. Two fundamental approaches have been used so far to calculate the shot noise contribution via numeric solution [78], [80] or via simulation [87], [88]. Mathematically, the shot noise free efficiency distribution 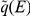 is convoluted with a shot noise kernel 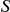 resulting in an efficiency distribution 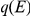 including the shot noise. This convolution
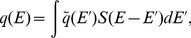
(21)broadens the underlying efficiency distribution 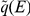 to 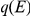. Because of this broadening, the reconstructed distance distribution 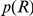 is narrowed when shot noise is taken into account. In analogy to image reconstruction from a de-focused image by inversion of the convolution with the appropriate image transfer function, the achieved accuracy and the ability to recover finer details of the original distance distribution are limited by the information loss due to convolution of the shot noise kernel 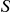 with the transfer function 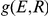, Eq. 14.

Since determining the shot noise kernel 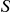 of an experimental BSD is non-trivial, the experimental shot noise (bursts 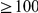) was included in the transfer function as follows. Each distance bin of the transfer matrix (columns in Fig. 12) was randomly sampled by 1200 bursts from the experimental BSD. The target efficiency for each burst was directly calculated from the transfer function (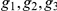) or randomly picked from the efficiency distribution (for the following refinement steps). According to the target efficiency, donor and acceptor photons were randomly generated, and the obtained burst efficiency was then recorded in the transfer function. Figure 12D and H illustrate the impact of an experimental shot noise (bursts 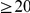) on transfer functions (Fig. 12C and G). Comparison of C and D illustrates, that after the inclusion of shot noise, the transfer function not uniquely defines an efficiency 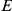 value for each distance 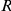, but instead an efficiency distribution. The here observed effect of the BSD on the transfer function is purely of stochastic origin, whereas a similar but independent effect will be seen in the following refinement level.

To motivate this level of refinement, recall that in all levels of refinement considered so far the full structure ensemble has been used to calculate appropriate averages for the orientation factor 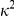. This approach implies the salient assumption that each single burst samples the same dye orientation distribution – which, however, holds true only if all components of the dye motion are much faster than the burst duration. As has been shown from the above comparison between measured efficiency distributions and those obtained from three different structure ensembles, there are slow components of the dye motion, which may render this salient assumption questionable. Our last level of refinement attempts to include the dominant effect of this limited dye orientation sampling within the transfer function. Note, however, that a rigorous treatment of this effect would require to go beyond the limits of the transfer function framework, and here is only achieved conceptually by our explicit hybrid MD/MC simulation approach.

At this refinement level, accordingly, the columns of the transfer matrix are formed from distance dependent transfer efficiency distributions 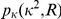 rather than single valued 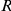-dependent averages 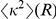, from which the transfer function 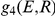 is derived as
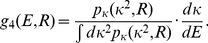
(22)


Here, the integral over 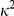 in the denominator of 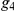 normalizes the probability distribution on the distances and 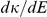 transforms 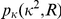 to 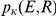. The normalized transfer function is obtained from orientation factor histograms for different distances from the MD trajectory ensemble, applying Eq. 2.

Figure 12E shows the transfer function resulting from the samples of our simulations. Notably, there is a distance dependent maximum efficiency due to the 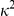 range from 0 to 4. The samples of 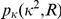 from our simulations each determine the efficiency samples of 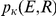. In experiments, however, efficiencies are determined using multiple photons. Thus, the efficiencies in the transfer function need to be averaged over multiple 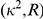 samples according to the BSD. Figure 12F shows this effect for a constant burst size of 5. As seen, this burst size dependent averaging introduces a narrowing of the transfer function, independent of the photon shot noise.

**Figure 14:**
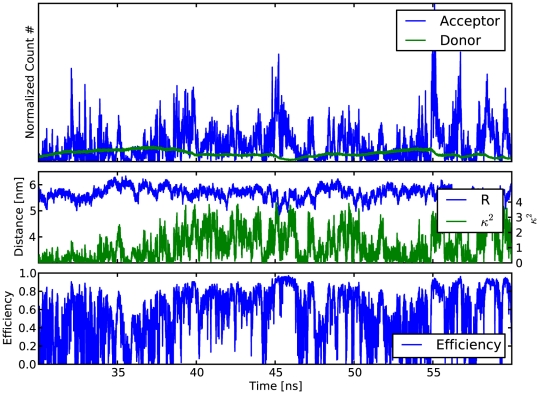
Time dependent photon emission along a single trajectory. Top: normalized acceptor (blue) and donor (green) photon count for time independent excitation probability. Mid: corresponding distance 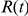 and orientation factor 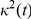. Bottom: resulting time dependent instantaneous efficiency.

Figure 14 motivates and illustrates the next level of refinement. Shown are the donor and acceptor photon counts from a trajectory, extensively sampled with photons created in our MC process. In high efficiency regions with efficiency values in the lower plot close to one (e.g., around 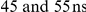), a marked depletion of donor and acceptor photon counts (dips in the green curve in the upper plot) is observed. As a result, also the mean intensity 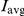 depends on 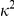 and 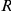 and thus affects the probability of obtaining a photon from a distinct 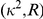 conformation. Thus, for each instant 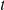, the orientation 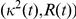 depends on the history of orientations within the fluorescence lifetime. Because in the construction of 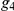, the probability distribution of dye orientations 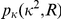 is only normalized at each distance, this memory is not described. By applying appropriate intensity weights, our last, most realistic transfer function 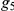 includes also this effect.
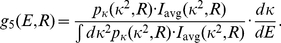
(23)


In our transfer function construction, the intensity of each 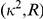 sample was determined by extensive photon sampling of our trajectories. Thereby, the adsorption events were equally distributed over the whole trajectory and the emission times of the photons was recorded. The 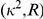 samples were then weighted according to their total emitted photon count. Notably, the samples are implicitly weighted according to their efficiency history in experiments. In Fig. 12G, a shift towards higher efficiencies as an effect of this weighting is seen. To reduce computationally expensive photon sampling of the trajectories, 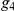 and 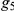 were calculated from 20 all-*trans* simulations only.

Applying this transfer function 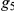 to our known distance distributions to asses the quality of approximations results in efficiency distributions only slightly different from the ones for 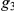 (Fig. 13 A, B). Nevertheless, as seen in Fig. 13, the high efficiencies in the experimental ensemble were reproduced better than for 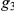. When using 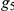 for the reconstruction of distances using the synthetic efficiencies, the best agreement with the distance distribution from the simulations was found (Fig. 13 E). Also, the reconstructed peak location using the experimental efficiencies is slightly closer to the peak from the simulations (Fig. 13 F).

Overall, in the experimental reconstruction, all distance distributions of different refinement levels are shifted towards lower distances in comparison to the simulation distance distribution. This agrees with the observation of low efficiency overestimation shown in Fig. 10.

These tests demonstrate, that a markedly improved reconstruction over the established approaches is achieved by inclusion of dye motion and photon statistics obtained by our hybrid simulation approach of simulated data. Further, by using step-by-step refined approximation levels for the transfer functions, a systematic improvement of the inverse distance reconstruction is achieved for the polyproline system.

### Conclusions

We have demonstrated that structural information on the dynamics of FRET dye pairs from MD simulations improves the reconstruction of distances and distance distributions from experimental FRET efficiency distributions over the usual 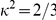 approximation, which assumes isotropic and uncorrelated distributions of the dye transition dipole orientations. A hybrid MC/MD method was developed and tested, which uses this structural information in combination with a Monte Carlo description of photon absorption, FRET-transfer, and emission, to calculate quantitative efficiency distributions. Based on the obtained good agreement with measured efficiency distributions of polyproline constructs, we have investigated several levels of approximation, resting on the particular relation of the different relevant time scales of the experiment and of the simulations. For the system at hand, this analysis revealed a previously unknown slow component of the dye movement. Our analysis further highlights that careful consideration of the time scales of the involved processes is crucial, and offers a framework that is flexible enough to capture the different time scale relationships expected for a broad range of systems. Unexpectedly, already for the simple polyproline system at hand, where the dyes are usually assumed to be sufficiently flexible to justify the established 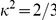 approximation, severe deviations were seen. Our results suggest that for FRET dye pairs attached to proteins or DNA/RNA complexes, the orientational dynamics are typically more restricted due to sterical hindrance and electrostatic interactions, a simulation approach like the one developed here is essential.

## Supporting Information

Figure S1
**Autocorrelation decay times of multiple parameters**. 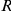 is the inter-dye distance, 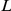 the chain end-to-end distance and 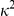 the orientation factor. The 3D, 2D and 2nd Legendre Polynomial of 2D (Anisotropy decay) was determined from Alexa 488. The decay here is from an all-*trans* polyproline-20 simulation.(EPS)

Figure S2 **Polyproline-30** ***cis*** **-isomer efficiencies.** For each isomer, the normalized probability is shown.
(EPS)

Video S1
**Distance and Orientation factor from simulations.** For illustration, a fragment of 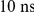 simulation time from polyproline-15 with the two dyes attached is shown as example for the dynamics. The box in the bottom shows the time dependent orientation and distance as well as the resulting FRET efficiency. The position in the time trace is shown as moving red bar in the box.(M4V)

Video S2 **FRET from Monte Carlo and simulation trajectories.** In addition to Video S1, exemplary excitation and de-excitation events are shown. Both competing pathways, de-excitation of donor, as well as the alternate pathway via FRET followed by acceptor de-excitation are displayed.
(M4V)

Text S1 (PDF)

Table S1 (PDF)
