# Supplementary material for: Structural Heterogeneity and Quantitative FRET Efficiency Distributions of Polyprolines through a Hybrid Atomistic Simulation and Monte Carlo Approach
Source: PLoS One. 2011 May 24;6(5):e19791. doi: 10.1371/journal.pone.0019791 (PMC3101224; doi:10.1371/journal.pone.0019791)
Supplement: Text S1 — (PDF) [file pone.0019791.s005.pdf]

Supplementary Materials

Cis-model

To calculate structural properties of the *cis*-simulations, the obtained average efficiencies were fitted to a simple geometrical model.

$$R_{\text{tot}} = \left(\frac{1}{E_{\text{tot}}} - 1\right)^{\frac{1}{6}} \cdot R_0 \tag{24}$$

$$R_A(i, x_0) = i \cdot \frac{R_{\text{tot}}}{20} - \frac{R_{\text{tot}}}{40} - x_0 \, [\forall i \in \{1, \ldots, 20\}] \tag{25}$$

$$R_D(i, x_0) = R_{\text{tot}} - R_A(i, x_0) \tag{26}$$

$$R_x(i, x_0, \alpha) = \sqrt{R_D^2 + R_A^2 + 2 \cdot R_D \cdot R_A \cos\alpha} \tag{27}$$

Its basis is a triangle, containing donor and acceptor arm lengths R_D and R_A (Eq. 25 and 26) from the *cis*-kink at the i-th position and the angle between the two arms α. The third side in the triangle is the end-to-end distance as dye separation R_x (Eq. 27). Additionally, an asymmetry x_0 is added to one and subtracted from the other arm length. The all-*trans* average efficiency is used to define the length of our chain R_{tot} according to Eq. 1 in our model. The position of the kink, and thus the length of the two arms, is determined by splitting R_{tot} into 20 segments and choosing the midpoint as kink. During the fit, the parameters α and x_0 are optimized.

Simulation box creation

First, the polyproline was aligned along the principal component of the terminal prolines (x-axis) and embedded in a box with a minimal distance of 1.5 nm to the boundaries. Then, the box vectors perpendicular to the principal component (y and z-direction) were symmetrized by applying either the larger one or a length of at least 6.2 nm to the y and z box vector length. This allows application of weak restraints to prevent rotation. Finally, the box vector in x-direction was enlarged by 1.8 nm to suppress interaction of the dyes with the periodic image of their counterpart (Fig. 2B).

Histograms

For efficiency histograms 50 histogram bins were used, evenly spaced from 0 to 1. Additionally, a random number in the range ± 0.00001 was added to all efficiency values. This prevents problems at the bin boundaries e.g. at 0.5, where all efficiencies with equal donor and acceptor ratio are put in bin # 25. In experimental setups, the raw data exhibits the same problems, however it is usually masked by the instrument corrections.

Persistence length calculation

Persistence length was determined from segment tangents to the chain. These tangents were defined by the vector $\text{C} - \alpha_i$ and $\text{C} - \alpha_i + (3 \cdot n)$ due to the periodicity of 3 in the polyproline II helix. The next segment corresponds to $n = 1$ and n up to $\frac{1}{3}$ of the chain segment count were considered. The cosines for all trajectory frames of each simulation and each valid n were calculated from which then, the persistence length was derived via Eq. 13.

Dye parameters

see dye-params-table.txt file
