# Supplementary material for: Structural Heterogeneity and Quantitative FRET Efficiency Distributions of Polyprolines through a Hybrid Atomistic Simulation and Monte Carlo Approach
Source: PLoS One. 2011 May 24;6(5):e19791. doi: 10.1371/journal.pone.0019791 (PMC3101224; doi:10.1371/journal.pone.0019791)
Supplement: Table S1 — (PDF) [file pone.0019791.s006.pdf]

Table S1. Point charge parameterization

Amino acid	OPLS Scaling Factor	StdErr
ALA	0.99	0.06
ARG	1.10	0.08
ASN	0.86	0.13
ASP	1.13	0.15
CYS	0.91	0.14
GLN	0.77	0.14
GLU	0.97	0.09
GLY	0.96	0.15
HIS	1.01	0.13
ILE	0.87	0.09
LEU	0.89	0.06
LYS	0.84	0.13
MET	0.89	0.08
PHE	0.74	0.14
PRO	0.55	0.18
SER	1.02	0.06
THR	0.93	0.10
TRP	0.74	0.15
TYR	0.78	0.14
VAL	0.76	0.14

Comparison between OPLS-AA [57] and DFT point charges of all 20 natural amino acids. Scaling factors with standard errors applied to the DFT charges in order to obtain the best fit for each amino acid. The mean scaling factor is 0.9.
